# Seeking adverse effects in systematic reviews of orthodontic interventions: protocol for a cross-sectional study

**DOI:** 10.1186/s13643-019-1000-1

**Published:** 2019-04-05

**Authors:** Pauline A. J. Steegmans, Shandra Bipat, Reint A. Meursinge Reynders

**Affiliations:** 1 Department of Orthodontics, Academisch Centrum Tandheelkunde Amsterdam (ACTA), University of Amsterdam, Gustav Mahlerlaan 3004, 1081 LA Amsterdam, The Netherlands; 2 Department of Radiology, Academic Medical Center, University of Amsterdam, Meibergdreef 9, 1105 AZ Amsterdam, The Netherlands; 3 Studio di ortodonzia, Via Matteo Bandello 15, 20123 Milan, Italy; 4 Department of Oral and Maxillofacial Surgery, Academic Medical Center, University of Amsterdam, Meibergdreef 9, 1105 AZ Amsterdam, The Netherlands

**Keywords:** Orthodontics, Reporting, Systematic review, Interventions, Adverse effect, Adverse event, Harm, Safety, Side effect, Patient-important outcomes

## Abstract

**Background:**

Before implementing healthcare interventions, clinicians need to weigh the beneficial and adverse effects of interventions. However, a large body of evidence has demonstrated that seeking and reporting of adverse effects is suboptimal in clinical trials and in systematic reviews of interventions. This cross-sectional study will investigate the status of this problem in orthodontics. This study will assess whether adverse effects were sought and whether findings related to adverse effects were reported in systematic reviews of orthodontic interventions in the five leading orthodontic journals and in the Cochrane Database of Systematic Reviews.

**Methods:**

Systematic reviews of clinical orthodontic interventions published between 01 August 2009 and 31 July 2019 in the five leading orthodontic journals and in the Cochrane Database will be included. Empty reviews will be excluded. The reporting of outcomes on adverse effects will not determine eligibility, i.e., reviews will not be excluded, because they did not report usable data. Study selection and data extraction will be conducted independently by two authors. Our primary outcome will be the prevalence of systematic reviews of orthodontic interventions that sought any findings related to adverse effects in the included studies. Additional prevalence statistics will be calculated on a series of items related to seeking of adverse effects in the eligible reviews. All statistics will be calculated for (1) all journals together, (2) the group of five orthodontic journals and the Cochrane Database of Systematic Reviews separately, and (3) each individual journal separately. Chi-square tests of independence will be used to compare these groups.

**Discussion:**

This study will assess whether adverse effects were sought in systematic reviews of orthodontic interventions. This knowledge is important, because reviews that present an incomplete picture on adverse effects can have unfavorable consequences for the end-users. Also not reporting that no adverse effects were assessed in eligible studies included in a systematic review can mislead pertinent stakeholders. Our findings could have policy implications for making judgments on accepting or rejecting an intervention systematic review for publication, for example, by directing editors and peer-reviewers to adopt the various items on adverse effects defined in the MECIR standards and in the PRISMA harm checklist.

**Electronic supplementary material:**

The online version of this article (10.1186/s13643-019-1000-1) contains supplementary material, which is available to authorized users.

## Background

Making balanced decisions on healthcare interventions requires reliable evidence on both their beneficial and adverse effects. In the Cochrane systematic reviews of interventions, it is therefore mandatory to seek both types of outcomes and include at least one undesirable outcome as a primary outcome measure [1, 2]. In both Cochrane and non-Cochrane reviews of orthodontic interventions, we will assess whether adverse effects were sought and whether findings related to adverse effects were reported.

Since its foundation in 1993, Cochrane has set the standard for medical research-synthesis publications [3]. Systematic reviews with or without meta-analyses are the core of such syntheses and are the foundations for evidence-based practice guidelines and policy. Cochrane reviews of interventions aim at including outcomes that are likely to be important for patients, clinicians, the general public, guideline developers, administrators, and policy makers [2]. Cochrane states: “It is critical that outcomes used to assess adverse effects as well as outcomes used to assess beneficial effects are among those addressed by a review” (chapter 5.4.1) [2]. This issue is important, because a balanced perspective of an intervention can only be obtained when both types of outcomes are assessed and reported with the same rigor. Cochrane has formulated the following definition of an adverse effect: “An adverse event for which the causal relation between the intervention and the event is at least a reasonable possibility” [4, 5]. We adopted Cochrane’s definitions of adverse effects, systematic reviews, and interventions reviews in this manuscript (Table 1) [4–6].

**Table 1:** Glossary of terms

Term	Definition
Systematic review	The Cochrane glossary [5] defines a systematic review as “A review of a clearly formulated question that uses systematic and explicit methods to identify, select, and critically appraise relevant research, and to collect and analyse data from the studies that are included in the review. Statistical methods (meta-analysis) may or may not be used to analyse and summarise the results of the included studies.”
Intervention review	Cochrane [6] defines an intervention review as follows: “Intervention reviews assess the benefits and harms of interventions used in healthcare and health policy.”
Orthodontic interventions	Orthodontic interventions refer to the use of any type of orthodontic appliances that are used to move teeth or change the jaw size or position for orthodontic purposes. These interventions also include appliances to maintain or stabilize the results of orthodontic treatment, for example retainers.
Adverse effect	Cochrane [4, 5] defines an adverse effect as “an adverse event for which the causal relation between the intervention and the event is at least a reasonable possibility.”

Numerous epidemiological studies have shown that adverse effects of interventions are often under-assessed or under-reported in primary research studies [7–11]. In addition, much information on adverse events remains unpublished and the number and range of these events are higher in unpublished compared to published versions of the same study [12]. To improve the reporting of harms in randomized trials, an extension of Consolidated Standards of Reporting Trials (CONSORT) Statement was developed [13]. The reporting of adverse events has improved over time since the publication of this extension, but was still suboptimal for a wide variety of clinical trials [9, 11, 14]. Systematic reviewers have an important role in bringing these issues to the foreground. However, epidemiological studies have shown that seeking and reporting adverse effects of interventions is also suboptimal in systematic reviews [15–18]. In 2016, the Preferred Reporting Items for Systematic reviews and Meta-Analyses (PRISMA) harm checklist [19] to improve harm reporting in systematic reviews was published, but the consequences of this checklist are still unknown.

In this study, we will assess whether adverse effects were sought and reported in systematic reviews of orthodontic interventions. We will scrutinize such reviews in the five leading orthodontic journals and those registered in the Cochrane Database of Systematic Reviews. Reporting on pain as a result of tooth movement and the various categories of known orthodontic adverse effects as defined by Preoteasa et al. [20] will be assessed in these reviews (Table 2). Scoping searches in the orthodontic literature confirmed the knowledge gaps on our research questions. Our pilot studies on intervention reviews of the Cochrane Database of Systematic Reviews and those published in the five leading orthodontic journals quantified these gaps and further showed the need to undertake this research study. Addressing our research objectives is crucial for patients, clinicians, researchers, policy makers, and research sponsors. These questions are particularly important, because systematic reviews are increasingly consulted by patients [21].

**Table 2:** Adverse effects hypothetically linked to orthodontic interventions [20]

Subgroup	Description
Local adverse effects	
Dental	• Crown: decalcifications, decays, tooth wear, enamel cracks and fractures; discolorations, deterioration of prosthetic crown (as fracturing a ceramic one during debonding) • Root: root resorption, early closure of root apex, ankylosis • Pulp: ischemia, pulpitis, necrosis
Periodontal	• Gingivitis, periodontitis, gingival recession or hypertrophy, alveolar bone loss, dehiscences, fenestrations, interdental fold, dark triangles
Temporomandibular joint	• Condylar resorption, temporomandibular dysfunction
Soft tissues of the oral and maxillofacial region	• Trauma (e.g., long archwires, headgear related), mucosal ulcerations or hyperplasia, chemical burns (e.g., etching related), thermal injuries (e.g., overheated burs), stomatitis, clumsy handling of dental instruments
Unsatisfactory treatment outcome	• Inadequate morpho-functional, esthetic or functional final result, relapse, failure to complete treatment due to treatment dropout
Systemic adverse effects	
Psychological	• Teasing, behavioral changes of patients and parents; discomfort associated with pain presence and esthetic look discontents during orthodontic appliance usage
Gastro-intestinal	• Accidental swallowing of small parts of the orthodontic device (tubes, brackets)
Allergies	• To nickel or latex
Cardiac	• Infective endocarditis
Chronic fatigue syndrome	
Cross infections	• From doctor to patient, patient to doctor, patient to patient

Permission to reproduce this table was obtained on 16 August 2018 from InTech’s Publishing Ethics and Legal Affairs Department

## Objectives

The main research question of this cross-sectional study is the following: “Do reviewers seek adverse effects in systematic reviews of orthodontic interventions?” To address this question, we have defined the following objectives:

- To calculate the prevalence of eligible systematic reviews of orthodontic interventions that defined seeking of adverse effects as a research objective of the review
- To calculate the prevalence of eligible systematic reviews of orthodontic interventions that sought any findings related to adverse effects in the included studies
- To calculate the prevalence of eligible systematic reviews of orthodontic interventions that considered and discussed (weighed) potential adverse effects of the intervention anywhere in the review
- To calculate the prevalence of each type of adverse effect sought in the review

## Methods

**Fig. 1:**
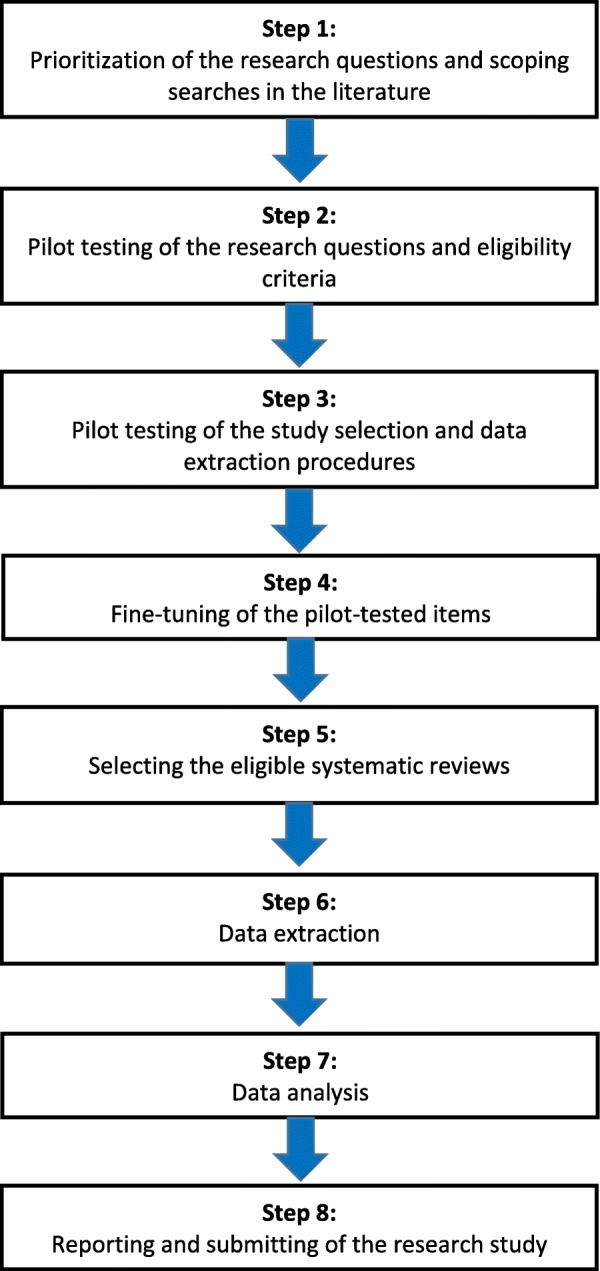
Flow diagram of the research methods

We used the Preferred Reporting Items for Systematic review and Meta-Analysis Protocols (PRISMA-P) 2015 statement as the guideline for reporting this protocol [22, 23]. The PRISMA-P checklist is included as Additional file 1. Figure 1 represents the flow diagram of our research methods. Our first step was to conduct scoping searches to identify knowledge gaps and prioritize research questions on seeking and reporting of adverse effects in systematic reviews of orthodontic interventions. Two reviewers (PS and RMR) subsequently conducted pilot tests to assess the validity of these questions and the research methods and to fine-tune them. The sample size for the pilot test was calculated a priori [24], and random numbers were generated to select pilot systematic reviews [25]. The procedures for our pilot tests are reported in Additional file 2. In the following sections, we presented our planned methods based on these pilot tests.

### Eligibility criteria

#### Study designs

- We will include systematic reviews of orthodontic interventions. The definition of a systematic review, an intervention review, and orthodontic interventions listed in the Glossary of terms will be used to assess whether a review is eligible (Table 1).
- We will exclude (1) non-interventional reviews such as “Methodology,” “Diagnostic,” “Qualitative,” and “Prognostic”; (2) rapid and scoping reviews; (3) systematic reviews that focus exclusively on adverse effects of interventions; and (4) systematic reviews of interventions that did not find any eligible studies (empty reviews).

#### Participants

- We will include systematic reviews on any type of patients undergoing orthodontic interventions, i.e., patients of any health status, sex, age, demographics, and socio-economic status.
- We will exclude (1) intervention reviews that focus exclusively on patients with congenital anomalies, for example, with cleft lip and palate, and (2) systematic reviews of animal or laboratory studies.

#### Interventions

- We will include the following: (1) Systematic reviews that assess the effects of clinical orthodontic interventions. Clinical orthodontic interventions refer to the use of any type of orthodontic appliances that are used to move teeth or change the jaw size or position for orthodontic purposes. (2) Systematic reviews of interventions with appliances to maintain or stabilize the results of orthodontic treatment, for example, retainers. (3) Systematic reviews of orthodontic interventions that compare the effects of orthodontic treatment with or without additional interventions such as pharmacological or small surgical procedures, e.g., periodontal or implant surgery.
- We will exclude (1) systematic reviews in which patients receive orthodontic treatment, but in which the effects of other interventions, e.g., periodontal surgery, are compared and not the effects of orthodontic interventions; (2) systematic reviews of interventions in which orthodontic appliances are specifically used for other purposes, e.g., changing jaw positions to treat respiration or temporomandibular disorders; and (3) systematic reviews of orthodontic interventions that included orthognathic surgery.
- No exclusion criteria will be applied to the characteristics of the operator who conducted the interventions.

#### Outcomes

- Any adverse effect of an orthodontic intervention scored at any endpoint or timing will be eligible.
- The effects of orthodontic interventions do not refer just to outcomes related to tooth and jaw size and positions, but also to broader outcomes such as periodontal health, esthetic changes, the health of the temporomandibular joint, patient health experiences, and economic issues associated with the intervention.
- The reporting of outcomes on adverse effects will not determine the eligibility of reviews for this cross-sectional study, i.e., reviews will not be excluded because they did not provide “usable” data [2].

#### Setting

- No exclusion criteria will be applied to the type of setting, e.g., university or private practice, etc., in which the interventions were conducted.

### Information sources

We will manually search eligible systematic reviews between 01 August 2009 and 31 July 2019 in the Cochrane Database of Systematic Reviews [26] and in the websites of the five leading orthodontic journals. We consulted the journal citation reports by Clarivate Analytics [27] to identify the five leading orthodontic journals based on their impact factor. Based on these reports, the following orthodontic journals were included: *European Journal of Orthodontics* [EJO], *American Journal of Orthodontics and Dentofacial Orthopedics* [AJODO], *Angle Orthodontist*, *The Korean Journal of Orthodontics*, and *Orthodontics and Craniofacial Research*. Recently launched orthodontic journals, i.e., covering less than 10 years of journal publication, will not be eligible. The first of August 2009 was chosen as the incept data for our searches, because it coincides with the launch of the Preferred Reporting Items for Systematic reviews and Meta-Analyses (PRISMA) statement and guidance on 21 July 2009 [28, 29].

### Study records

#### Data management

- All study selection and data extraction procedures will be conducted by two authors (PS and RMR) independently.
- Our pilot tests were also used to train both reviewers in applying our methods consistently and to calibrate them [23].
- Disagreement on the eligibility of a paper or the extraction of data will be resolved through (1) discussions between reviewers, (2) rereading the pertinent paper, or (3) contacting its authors by email [28]. Persistent disagreements will be resolved through the consultation of a methodologist (SB).
- All eligible systematic reviews will be downloaded as PDFs, and all data will be extracted to an Excel spreadsheet [30].

#### Selection process

- All titles and abstracts will be screened for eligibility in the websites of the five orthodontic journals. We will search the section “Dentistry and Oral health” for eligible reviews in the Cochrane Database of Systematic Reviews [26].
- When updates of reviews are identified, we will only consider the latest version.
- Authors suspect of multiple publications of the same systematic review will be contacted by email. We plan to consider the first publication, but this decision will be weighed on a case-by-case basis. Our rationale for these decisions will be reported in the completed study.
- A PRISMA flow diagram will illustrate our selection procedures [28, 29].
- All eligible and excluded systematic reviews will be presented in tables. The rationale for exclusion will be listed for each excluded review.

#### Data collection process

- Eligible studies and their pertinent supplemental files will be merged into binder PDFs, and multiple search terms will be applied to facilitate data extraction [31, 32].
- We consulted various articles on adverse effects [4, 13, 18, 19, 33, 34] and thesauri to develop these search terms. A table with all search terms is listed in Additional file 3.
- All pertinent data items will be extracted using our pilot tested data collection forms. These forms are presented in Additional file 4 and incorporate all our research questions. We consulted the PRISMA [28, 29] and the PRISMA-P [22, 23] checklists to develop these data collection forms.
- Criteria for scoring the pertinent data items are defined in these forms.
- We will search the entire eligible review, i.e., the text, tables, figures, and supplemental files. The plain language summary in eligible Cochrane systematic reviews will not be scrutinized for data items.
- Modifications made in the collection forms during data extraction will be reported in the section “Differences between the protocol and review” together with the rationale for these changes.

#### Scoring adverse effects of orthodontic interventions

- We will adopt a priori the various categories of known orthodontic adverse effects as defined by Preoteasa et al. [20], which were divided into two main types: local and systemic, with their pertinent subtypes (Table 2).
- We will also consider pain as a result of tooth movement and additional adverse effects of orthodontic interventions that are identified post hoc, i.e., during data extraction, and are not listed in Table 2. We will explain the rationale for including specific additional effects as adverse and will produce a framework for categorizing them.
- Ambiguous outcomes that could be interpreted as either beneficial or adverse will not be scored as “adverse.” We will also present the rationale for this score. Ambiguous outcomes will only be scored as adverse when the authors of the pertinent review define these outcomes as such.

### Outcomes and statistical analyses

**Fig. 2:**
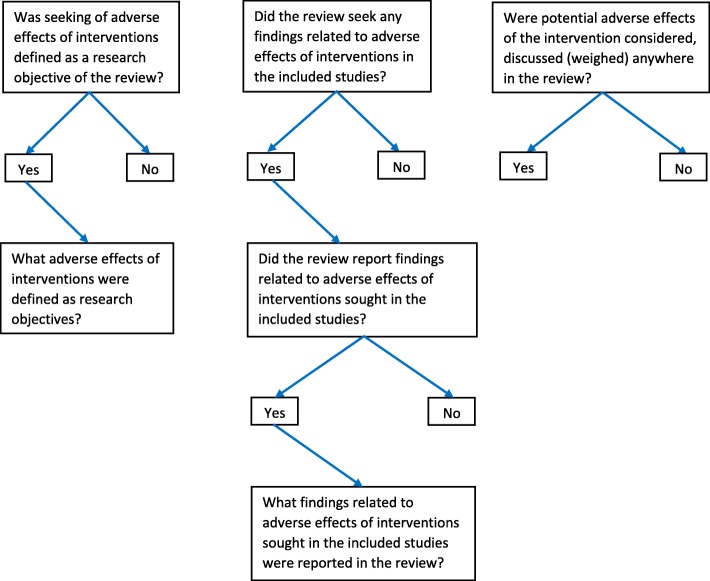
Flow diagram “seeking adverse effects of interventions in systematic reviews of orthodontic interventions”

- All research questions are presented in flow diagrams (Fig. 2).
- All planned outcomes are presented in a summary of findings table (Table 3).
- All prevalence data will be calculated and reported with their 95% confidence levels.
- Prevalence statistics will be calculated for (1) all journals together, (2) the group of five orthodontic journals and the Cochrane Database of Systematic Reviews separately, and (3) each individual journal separately. Comparisons between these statistics will be calculated. These statistics will be compared with chi-square tests of independence. We will report the value of chi-square, the degrees of freedom (*df*), and the *p* value. A *p* value of < 0.05 will be considered to be statistically significant. We will use Stata software (Stata Corporation, College Station, TX, USA) version 15 for all the statistical analyses [35].
- We will report all outcomes that will be introduced or eliminated post hoc together with the rationale for inclusion or exclusion.

**Table 3:** Summary of findings

Description of outcomes from the main text	Statistic
The number of retrieved systematic reviews	Number
The prevalence of eligible systematic reviews of orthodontic interventions	Prevalence
The prevalence of eligible systematic reviews of orthodontic interventions that defined seeking of adverse effects of interventions as a research objective of the review	Prevalence
The prevalence of eligible systematic reviews of orthodontic interventions that sought any findings related to adverse effects of interventions in the included studies	Prevalence
The prevalence of eligible systematic reviews of orthodontic interventions that reported findings related to the adverse effects of interventions sought in the included studies	Prevalence
The prevalence of eligible systematic reviews of orthodontic interventions that considered, discussed (weighed) potential adverse effects of the intervention anywhere in the review	Prevalence
The prevalence of each type of adverse effect of interventions defined in the objectives of the review	Prevalence
The prevalence of each type of adverse effect of interventions sought in the review	Prevalence

All prevalence data will be presented with their 95% confidence intervals

### Reporting of the research study and data management

- We will adopt The Strengthening the Reporting of Observational Studies in Epidemiology (STROBE) Statement as the guideline for reporting the completed cross-sectional study [36].
- We prepared a data management plan for the long-term storage of our research data [37]. This plan guarantees that (1) all our project data will be made freely available and (2) our submitted article will be accompanied by additional files with all raw data of the completed study or with a link to a repository where these files will be deposited. In the latter case, we will register our repository in the Registry of Research Data Repositories [38]. (3) Our project data will be presented in a format that permits other scientists to understand, cite, and reuse the data. (4) Sensitive data will be protected. (5) Our data management plan will be frequently reassessed and updated if necessary [37, 38].

### Differences between the protocol and the completed study

All differences between the protocol and the final research study will be reported together with the rationale for these changes. We will also present the consequences of these modifications on the magnitude, direction, and validity of the outcomes [39].

## Discussion

### Strengths

We point at four key strengths of this research study. First, extensive scoping searches and pilot studies were conducted to fine-tune our research questions and procedures. Our pilot studies also confirmed the importance of our research questions. Second, the research team consisted of two topic experts (PS and RMR) and two methodologists (RMR and SB). Third, all study selection and data extraction procedures were conducted by two operators (PS and RMR) independently. Fourth, this study will permit reproducibility, because we will publish the protocol a priori and all raw data of the completed study will be reported in additional files or will be deposited in an open access repository [37, 40].

### Limitations

The limitations of this research study include the following: (1) It does not cover all journals that have published orthodontic intervention systematic reviews, but only a subgroup, i.e., those published in the five leading orthodontic journals and in the Cochrane Database of Systematic Reviews. However, we expect that the choice of this subgroup of the leading orthodontic literature will produce outcomes that will underestimate the true severity of the problem. (2) Including only systematic reviews of orthodontic interventions published in the last 10 years could introduce the risk of publication bias. However, we chose this period, because it will represent the actual knowledge status on assessing adverse effects in orthodontic intervention systematic reviews. Further, this period coincides with the launch in 2009 of the PRISMA reporting checklist, which is an important update on how to report items in systematic reviews [28, 29].

### Importance and beneficiaries

In this research study, we will assess whether adverse effects were sought and reported in both Cochrane and non-Cochrane systematic reviews of orthodontic interventions. This is important, because of the following: (1) The validity of the findings of systematic reviews of interventions depends on a balanced presentation of both the benefits and adverse effects of the intervention [19]. (2) There is a large body of evidence that has demonstrated that seeking and reporting of adverse effects is suboptimal in a wide variety of clinical trials [7–11]. Systematic reviewers can have a crucial role as whistle blowers by bringing these knowledge gaps to the foreground. However, their position can also be damaging, because reviews that present an incomplete picture on these gaps can have unfavorable consequences for the end-users. For example, not reporting that no adverse effects were assessed in eligible studies included in a systematic review can mislead readers.

Our findings could have policy implications for making judgments on accepting or rejecting a systematic reviews of orthodontic interventions for publication, for example, by directing editors and peer-reviewers to adopt the various items on adverse effects defined in the Methodological Expectations of Cochrane Intervention Reviews (MECIR) standards [1] and the PRISMA harm checklist [19]. Patients, clinicians, researchers, editors, peer-reviewers, guideline developers, policy makers, and research funders will all benefit from the findings of this research study.

## Additional files

Additional file 1: Checklist for the Preferred Reporting Items for Systematic review and Meta-Analysis Protocols (PRISMA-P) 2015 statement. (DOCX 33 kb)

Additional file 2: Pilot tests. (DOCX 21 kb)

Additional file 3: Search terms and their derivatives. (DOCX 15 kb)

Additional file 4: Data collection forms. (DOCX 16 kb)

## Abbreviations

EQUATOR Network: Enhancing the Quality and Transparency Of health Research Network
MECIR: Methodological Expectations of Cochrane Intervention Reviews
PRISMA: Preferred Reporting Items for Systematic reviews and Meta-Analyses
PRISMA-P: Preferred Reporting Items for Systematic review and Meta-Analysis-Protocols
