# Supplementary material for: Seeking adverse effects in systematic reviews of orthodontic interventions: protocol for a cross-sectional study
Source: Syst Rev. 2019 Apr 5;8:89. doi: 10.1186/s13643-019-1000-1 (PMC6449933; doi:10.1186/s13643-019-1000-1)
Supplement: Supplementary file 2 — Pilot tests. (DOCX 21 kb) [file 13643_2019_1000_MOESM2_ESM.docx]

**Additional file 2. Pilot tests**

Eligible systematic reviews of orthodontic interventions published in the eligible journals were used for our pilot tests and were stratified evenly over the eligible years and journals. We calculated the required sample size of our pilot study based on the probability of Yes scores for our primary research question ‘Did the review seek any findings related to adverse effects of interventions in the included studies?

To calculate this sample size we used the following equation [24]:

n = $\frac{ln(1-ϒ)}{\ln(1-\pi)}$

n = the sample size for the pilot study

ϒ = the threshold of confidence (95%)

π = the probability of a ‘Yes’ score

We calculated the sample size separately for systematic reviews of orthodontic interventions in the Cochrane Database of Systematic Reviews and those published in the 5 leading orthodontic journals. We used the probabilities of the Yes scores identified during our scoping searches as our benchmark. These probabilities were respectively 1 and 0.25. Based on these probabilities we calculated the required sample sizes of respectively 1 and 10.4. To play it safe we planned to include at least 2 Cochrane reviews and 12 orthodontic reviews in our pilot study. We planned increasing the size of the pilot study when the probability of yes scores for the pertinent review types would be inferior in the pilot studies compared to those identified in the scoping searches. We used random number generator software [25] to select the pilot reviews. We searched the first eligible review published in the random issue number of the pertinent journal for the years 2009, 2010, and 2011. If no eligible review was identified for that issue, we searched the first eligible review published in subsequent issues of the stratified journal subgroup. If no eligible reviews were identified for that period, we searched in the issues published prior to the random issue number and back to the starting issue of the stratified journal subgroup. We applied this strategy for the 3 stratified subgroups, i.e., 2009-2011, 2012-2014, 2015-2017 (Table). The prevalence of eligible systematic reviews that sought any findings related to adverse effects in the included studies was 25% (3/12) for the orthodontic journals and 100% (2/2) for reviews identified in the Cochrane Database of Systematic reviews.

**Table. Systematic reviews of orthodontic interventions selected for the pilot test**

| **Journal** | **Year and number of issues** | **Random issue number** | **Selected systematic review** |
| --- | --- | --- | --- |
| Cochrane Database of Systematic reviews | 2009, 2010, and 2011 (28 issues) | 24 | No reference identified |
| Cochrane Database of Systematic reviews | 2012, 2013, and 2014  (36 issues) | 31 | [Agostino P](https://www.ncbi.nlm.nih.gov/pubmed/?term=Agostino%20P%5BAuthor%5D&cauthor=true&cauthor_uid=25104166), [Ugolini A](https://www.ncbi.nlm.nih.gov/pubmed/?term=Ugolini%20A%5BAuthor%5D&cauthor=true&cauthor_uid=25104166), [Signori A](https://www.ncbi.nlm.nih.gov/pubmed/?term=Signori%20A%5BAuthor%5D&cauthor=true&cauthor_uid=25104166), [Silvestrini-Biavati A](https://www.ncbi.nlm.nih.gov/pubmed/?term=Silvestrini-Biavati%20A%5BAuthor%5D&cauthor=true&cauthor_uid=25104166), [Harrison JE](https://www.ncbi.nlm.nih.gov/pubmed/?term=Harrison%20JE%5BAuthor%5D&cauthor=true&cauthor_uid=25104166), [Riley P](https://www.ncbi.nlm.nih.gov/pubmed/?term=Riley%20P%5BAuthor%5D&cauthor=true&cauthor_uid=25104166).  Orthodontic treatment for posterior crossbites.  [Cochrane Database Syst Rev.](https://www.ncbi.nlm.nih.gov/pubmed/?term=Orthodontic+treatment+for+posterior+crossbites+Paola+Agostino) 2014 Aug 8;(8):CD000979. |
| Cochrane Database of Systematic reviews | 2015, 2016, and 2017 (36 issues) | 12 | Littlewood SJ, Millett DT, Doubleday B, Bearn DR, Worthington HV. Retention procedures for stabilising tooth position after treatment with orthodontic braces Cochrane Database Syst Rev. 2016 Jan 29;(1):CD002283. |
| European Journal of Orthodontics | 2009, 2010, and 2011 (18 issues) | 10 | Naoumova J, Kurol J, Kjellberg H. A systematic review of the interceptive treatment of palatally displaced maxillary canines. Eur J Orthod. 2011 Apr;33(2):143-9. |
| European Journal of Orthodontics | 2012, 2013, and 2014  (18 issues) | 5 | Zuccati G, Casci S, Doldo T, Clauser C. Expansion of maxillary arches with crossbite: a systematic review of RCTs in the last 12 years. Eur J Orthod. 2013 Feb;35(1):29-37. |
| European Journal of Orthodontics | 2015, 2016, and 2017 (18 issues) | 3 | [Koretsi V](https://www.ncbi.nlm.nih.gov/pubmed/?term=Koretsi%20V%5BAuthor%5D&cauthor=true&cauthor_uid=25398303), [Zymperdikas VF](https://www.ncbi.nlm.nih.gov/pubmed/?term=Zymperdikas%20VF%5BAuthor%5D&cauthor=true&cauthor_uid=25398303), [Papageorgiou SN](https://www.ncbi.nlm.nih.gov/pubmed/?term=Papageorgiou%20SN%5BAuthor%5D&cauthor=true&cauthor_uid=25398303), [Papadopoulos MA](https://www.ncbi.nlm.nih.gov/pubmed/?term=Papadopoulos%20MA%5BAuthor%5D&cauthor=true&cauthor_uid=25398303). Treatment effects of removable functional appliances in patients with Class II malocclusion: a systematic review and meta-analysis. [Eur J Orthod.](https://www.ncbi.nlm.nih.gov/pubmed/?term=Treatment+effects+of+removable+functional+appliances+in+patients+with+Class+II+malocclusion%3A+a+systematic+review+and+meta-analysis++Vasiliki+Koretsi) 2015 Aug;37(4):418-34. |
| American Journal of Orthodontics and Dentofacial Orthopedics | 2009, 2010, and 2011 (36 issues) | 18 | Chen SS, Greenlee GM, Kim JE, Smith CL, Huang GJ. Systematic review of self-ligating brackets. Am J Orthod Dentofacial Orthop. 2010 Jun;137(6):726.e1-726.e18; discussion 726-7. |
| American Journal of Orthodontics and Dentofacial Orthopedics | 2012, 2013, and 2014  (36 issues) | 35 | Yang X, Li C, Bai D, Su N, Chen T, Xu Y, Han X.  [Treatment effectiveness of Fränkel function regulator on the Class III malocclusion: a systematic review and meta-analysis.](https://www.ncbi.nlm.nih.gov/pubmed/25085296)  Am J Orthod Dentofacial Orthop. 2014 Aug;146(2):143-54. |
| American Journal of Orthodontics and Dentofacial Orthopedics | 2015, 2016, and 2017 (36 issues) | 9 | [Nucera R](https://www.ncbi.nlm.nih.gov/pubmed/?term=Nucera%20R%5BAuthor%5D&cauthor=true&cauthor_uid=27131242), [Lo Giudice A](https://www.ncbi.nlm.nih.gov/pubmed/?term=Lo%20Giudice%20A%5BAuthor%5D&cauthor=true&cauthor_uid=27131242), [Rustico L](https://www.ncbi.nlm.nih.gov/pubmed/?term=Rustico%20L%5BAuthor%5D&cauthor=true&cauthor_uid=27131242), [Matarese G](https://www.ncbi.nlm.nih.gov/pubmed/?term=Matarese%20G%5BAuthor%5D&cauthor=true&cauthor_uid=27131242), [Papadopoulos MA](https://www.ncbi.nlm.nih.gov/pubmed/?term=Papadopoulos%20MA%5BAuthor%5D&cauthor=true&cauthor_uid=27131242), [Cordasco G](https://www.ncbi.nlm.nih.gov/pubmed/?term=Cordasco%20G%5BAuthor%5D&cauthor=true&cauthor_uid=27131242). Effectiveness of orthodontic treatment with functional appliances on maxillary growth in the short term: A systematic review and meta-analysis. [Am J Orthod Dentofacial Orthop.](https://www.ncbi.nlm.nih.gov/pubmed/27131242) 2016 May;149(5):600-611.e3. |
| Angle Orthodontist | 2009, 2010, and 2011 (18 issues) | 6 | [Leonardi R](https://www.ncbi.nlm.nih.gov/pubmed/?term=Leonardi%20R%5BAuthor%5D&cauthor=true&cauthor_uid=19852663), [Annunziata A](https://www.ncbi.nlm.nih.gov/pubmed/?term=Annunziata%20A%5BAuthor%5D&cauthor=true&cauthor_uid=19852663), [Licciardello V](https://www.ncbi.nlm.nih.gov/pubmed/?term=Licciardello%20V%5BAuthor%5D&cauthor=true&cauthor_uid=19852663), [Barbato E](https://www.ncbi.nlm.nih.gov/pubmed/?term=Barbato%20E%5BAuthor%5D&cauthor=true&cauthor_uid=19852663).  Soft tissue changes following the extraction of premolars in nongrowing patients with bimaxillary protrusion. A systematic review. [Angle Orthod.](https://www.ncbi.nlm.nih.gov/pubmed/?term=Soft+Tissue+Changes+Following+the+Extraction+of+Premolars+in+Nongrowing+Patients+With+Bimaxillary+Protrusion) 2010 Jan;80(1):211-6. |
| Angle Orthodontist | 2012, 2013, and 2014  (18 issues) | 4 | [Feng X](https://www.ncbi.nlm.nih.gov/pubmed/?term=Feng%20X%5BAuthor%5D&cauthor=true&cauthor_uid=22458766), [Li J](https://www.ncbi.nlm.nih.gov/pubmed/?term=Li%20J%5BAuthor%5D&cauthor=true&cauthor_uid=22458766), [Li Y](https://www.ncbi.nlm.nih.gov/pubmed/?term=Li%20Y%5BAuthor%5D&cauthor=true&cauthor_uid=22458766), [Zhao Z](https://www.ncbi.nlm.nih.gov/pubmed/?term=Zhao%20Z%5BAuthor%5D&cauthor=true&cauthor_uid=22458766), [Zhao S](https://www.ncbi.nlm.nih.gov/pubmed/?term=Zhao%20S%5BAuthor%5D&cauthor=true&cauthor_uid=22458766), [Wang J](https://www.ncbi.nlm.nih.gov/pubmed/?term=Wang%20J%5BAuthor%5D&cauthor=true&cauthor_uid=22458766).  Effectiveness of TAD-anchored maxillary protraction in latemixed dentition. [Angle Orthod.](https://www.ncbi.nlm.nih.gov/pubmed/?term=Effectiveness+of+TAD-anchored+maxillary+protraction+in+late+mixed) 2012 Nov;82(6):1107-14. |
| Angle Orthodontist | 2015, 2016, and 2017 (18 issues) | 13 | Diar-Bakirly S, Feres MF, Saltaji H, Flores-Mir C, El-Bialy T. Effectiveness of the transpalatal arch in controlling orthodontic anchorage in maxillary premolar extraction cases: A systematic review and meta-analysis.Angle Orthod. 2017 Jan;87(1):147-158. |
| The Korean Journal of Orthodontics | 2009, 2010, and 2011 (18 issues) | 7 | No reviews identified in all 18 issues |
| The Korean Journal of Orthodontics | 2012, 2013, and 2014  (18 issues) | 14 | No reviews identified in all 18 issues |
| The Korean Journal of Orthodontics | 2015, 2016, and 2017 (18 issues) | 7 | Papageorgiou SN, Höchli D, Eliades T. Outcomes of comprehensive fixed appliance orthodontic treatment: A systematic review with meta-analysis and methodological overview. Korean J Orthod. 2017 Nov; 47(6): 401–413. |
| Orthodontics and Craniofacial Research | 2009, 2010, and 2011 (12 issues) | 1 | Several reviews were identified, but none were eligible |
| Orthodontics and Craniofacial Research | 2012, 2013, and 2014  (12 issues) | 3 | Cordasco G, Matarese G, Rustico L, Fastuca S, Caprioglio A, Lindauer SJ, Nucera R. Efficacy of orthopedic treatment with protraction facemask on skeletal Class III malocclusion: a systematic review and meta-analysis. Orthod Craniofac Res. 2014 Aug;17(3):133-43. |
| Orthodontics and Craniofacial Research | 2015, 2016, and 2017 (12 issues) | 1 | [Al-Saleh MA](https://www.ncbi.nlm.nih.gov/pubmed/?term=Al-Saleh%20MA%5BAuthor%5D&cauthor=true&cauthor_uid=26260422), [Alsufyani N](https://www.ncbi.nlm.nih.gov/pubmed/?term=Alsufyani%20N%5BAuthor%5D&cauthor=true&cauthor_uid=26260422), [Flores-Mir C](https://www.ncbi.nlm.nih.gov/pubmed/?term=Flores-Mir%20C%5BAuthor%5D&cauthor=true&cauthor_uid=26260422), [Nebbe B](https://www.ncbi.nlm.nih.gov/pubmed/?term=Nebbe%20B%5BAuthor%5D&cauthor=true&cauthor_uid=26260422), [Major PW](https://www.ncbi.nlm.nih.gov/pubmed/?term=Major%20PW%5BAuthor%5D&cauthor=true&cauthor_uid=26260422).  Changes in temporomandibular joint morphology in class II patients treated with fixed mandibular repositioning and evaluated through 3D imaging: a systematic review. [Orthod Craniofac Res.](https://www.ncbi.nlm.nih.gov/pubmed/?term=Changes+in+temporomandibular+joint+morphology+in+class+II+patients+treated+with+fixed+mandibular+repositioning+and+evaluated+through+3D+imaging%3A+a+systematic+review) 2015 Nov;18(4):185-201. |
