# Supplementary material for: Seeking adverse effects in systematic reviews of orthodontic interventions: protocol for a cross-sectional study
Source: Syst Rev. 2019 Apr 5;8:89. doi: 10.1186/s13643-019-1000-1 (PMC6449933; doi:10.1186/s13643-019-1000-1)
Supplement: Supplementary file 3 — Search terms and their derivatives. (DOCX 15 kb) [file 13643_2019_1000_MOESM3_ESM.docx]

**Additional file 3. Search terms and their derivatives**

**Table. Search terms and their derivatives**

| **Search terms and their derivatives** | **Search terms for searching multiple words in a PDF** |
| --- | --- |
| “adverse” | ADVERSE, Adverse, adverse |
| “effect”, “effects” | EFFECT, Effect, effect |
| “reaction”, “reactions” | REACTION, Reaction, reaction |
| “complication”, “complications”, “complicated”, “complicating” | COMPLICAT, Complicat, complicat |
| “harm”, “harms”, “harmful” | HARM, Harm, harm |
| “risk”, “risks”, “risky” | RISK, Risk, risk |
| “safe”, “safety” | SAFE, Safe, safe |
| “side” | SIDE, Side, side |
| “toxic”, “toxicity” | TOXIC, Toxic, toxic |
| “benefit”, “benefits” | BENEFIT, Benefit, benefit |
| “result”, “results” | RESULT, Result, result |
| “finding”, “findings” | FINDING, Finding, finding |
| “outcome”, “outcomes” | OUTCOME, Outcome, outcome |
| “limitation”, “limitations”, limit | LIMIT, Limit, limit |
| “damage”, “damages”, “damaging” | DAMAGE, Damage, damage |
| “data” | DATA, Data, data |
| “information” | INFO, Info, info |
| “conflict”, “conflicts”, “conflicting” | CONFLICT, Conflict, conflict |
| “negative” | NEGATIVE, Negative, negative |
| “detrimental” | DETRIMENTAL, Detrimental, detrimental |
| “disadvantage”, “disadvantages”, “disadvantageous” | DISADVAN, Disadvan, disadvan |
| “down” | DOWN, Down, down |
| “injury”, “injuries”, “injured”, “injurious” | INJUR, Injur, injur |
| “byproduct”, “byproducts” | BYPRODUCT, Byproduct, byproduct |
| “collateral” | COLLATERAL, Collateral, collateral |
| “unfavorable”, “unfavourable” | UNFAVO, Unfavo, unfavo |
| “destructive” | DESTRUCT, Destruct, destruct |
| “unsafe” | UNSAFE, Unsafe, unsafe |
| “undesired”, “undesirable” | UNDESIR, Undesir, undesir |
| “recommend”, “recommendation”, “recommending” | RECOMMEND, Recommend, recommend |
| “emergency”, “emergencies” | EMERGEN, Emergen, emergen |
