# Supplementary material for: Seeking adverse effects in systematic reviews of orthodontic interventions: protocol for a cross-sectional study
Source: Syst Rev. 2019 Apr 5;8:89. doi: 10.1186/s13643-019-1000-1 (PMC6449933; doi:10.1186/s13643-019-1000-1)
Supplement: Supplementary file 4 — Data collection forms. (DOCX 16 kb) [file 13643_2019_1000_MOESM4_ESM.docx]

**Additional file 4. Data collection forms**

**Table. Data collection forms***

| **Items for the main manuscript** | **Description** |
| --- | --- |
| Journal | List the pertinent journal |
| Year | Year of publication |
| Binder page number | List the binder page number |
| Reference | List full reference (Authors, Title, Journal) |
| Is the article a systematic review? | Answer: Yes/No  Consider definition of a systematic review |
| What type of systematic review? | List the type of systematic review.  Consider different types of systematic reviews.  When the publication is not an intervention systematic review describe what type it is or could be and classify. Types of systematic reviews will receive a final classification during the discussions between operators. |
| Were orthodontic interventions assessed? | Answer: Yes/No  Consider the definition of orthodontic interventions. |
| What was the orthodontic intervention? | List the type of orthodontic intervention  NA: When the article is not a systematic review or not a systematic review of interventions. |
| Is the systematic review eligible? | Answer: Yes/No  Yes: The article is a systematic review of an orthodontic intervention.  No: The article is not a systematic review of an orthodontic intervention.  No: The article is a systematic review of an orthodontic intervention, but focusses exclusively on its adverse effects. |
| Page and potential comments** | Present the pertinent pages of reference for scoring the previous items and list the potential comments. |
| Was seeking of adverse effects of interventions defined as a research objective of the review? | Answer: Yes/No  Yes: When seeking of adverse effects of interventions was defined as a research objective or as a research question or when adverse effects were predefined a priori as outcomes to assess.  No: Seeking of adverse effects of interventions was not defined as a research objective or as a research question or when adverse effects were not predefined a priori as outcomes to assess. |
| What adverse effects of interventions were defined as research objectives? | Answer: List adverse effects/NA  List all adverse effects of interventions that the reviewers defined as research objectives.  NA: When the following question was answered with a ‘No’: ‘Was seeking of adverse effects of interventions defined as a research objective of the review?’ |
| Did the review seek any findings related to adverse effects of interventions in the included studies? | Answer: Yes/No  Yes: Any findings related to adverse effects of interventions in the included studies were sought by the reviewers.  Seeking any findings related to adverse effects of interventions in the included studies refers to reporting anywhere in the review (except in the Abstract) that such adverse effects in the included studies were sought.  Yes: Yes is also scored when reviewers only reported findings related to adverse effects of interventions in the included studies, but did not report that they actually sought them or planned to seek them. For example ‘Yes’ will be scored when outcomes on adverse effects of interventions in the included studies were reported in the review, but were not defined as objectives of the review.  Yes: Yes is also scored when the reviewers reported that they planned to seek (for example in the research objectives) findings related to adverse effects of interventions in the included studies, but did not report on these findings.  No: Findings related to adverse effects of interventions in the included studies were not sought by the reviewers. |
| Did the review report findings related to adverse effects of interventions sought in the included studies? | Answer: Yes/No/NA  Yes: The review reported findings related to adverse effects of interventions sought in the included studies.  ‘Yes’ is also scored when the review reported that no findings on adverse effects of interventions in the included studies were identified.  No: The review did not report any findings related to adverse effects of interventions sought in the included studies.  NA: When the following question was answered with a No: ‘Did the review seek any findings related to adverse effects of interventions in the included studies?’ |
| What findings related to adverse effects of interventions sought in the included studies were reported in the review? | Answer: List of adverse effects/NA  List all findings related to adverse effect(s) of interventions that were identified in the included studies and reported in the review.  NA: When the following question was answered with a ‘No’: ‘Did the review seek any findings related to adverse effects of interventions in the included studies?’ |
| Rationale for assigning an effect as 'adverse' or 'not adverse' (In case of additional or ambivalent adverse effects) | Answer: Present the rationale for assigning an effect as 'adverse' or 'not adverse' (In case of additional or ambivalent adverse effects) |
| Page and potential comments** | Present the pertinent pages of reference for scoring the previous items and list the potential comments. |
| Were potential adverse effects of the intervention considered, discussed (weighed) anywhere in the review? | Answer: Yes/No  Yes: Potential adverse effect(s) of interventions in the included studies were sought and reported by the reviewers. ‘Yes’ is also scored when potential adverse effect(s) of interventions were not sought, but only considered, discussed (weighed) anywhere in the review.  ‘No’ is scored when potential adverse effects of the intervention were not considered, discussed (weighed) anywhere in the review. |
| Rationale for assigning an effect as 'adverse' or 'not adverse' (In case of additional or ambivalent adverse effects) | Answer: Present the rationale |
| Page and potential comments** | Present the pertinent pages of reference for scoring the previous items and list the potential comments. |

*To address our research question we will not consider what was reported regarding this question in the abstract and in the protocol of the review.

******When referring to a particular page in the systematic review, we will use the page number of the systematic review and not the number in the binder document.
